# Supplementary material for: PRG3 and PRG5 C-Termini: Important Players in Early Neuronal Differentiation
Source: Int J Mol Sci. 2022 Oct 27;23(21):13007. doi: 10.3390/ijms232113007 (PMC9657456; doi:10.3390/ijms232113007)
Supplement: Supplementary file 1 [file ijms-23-13007-s001.zip › ijms-1924038-supplementary.pdf]

Supplementary Figure S1

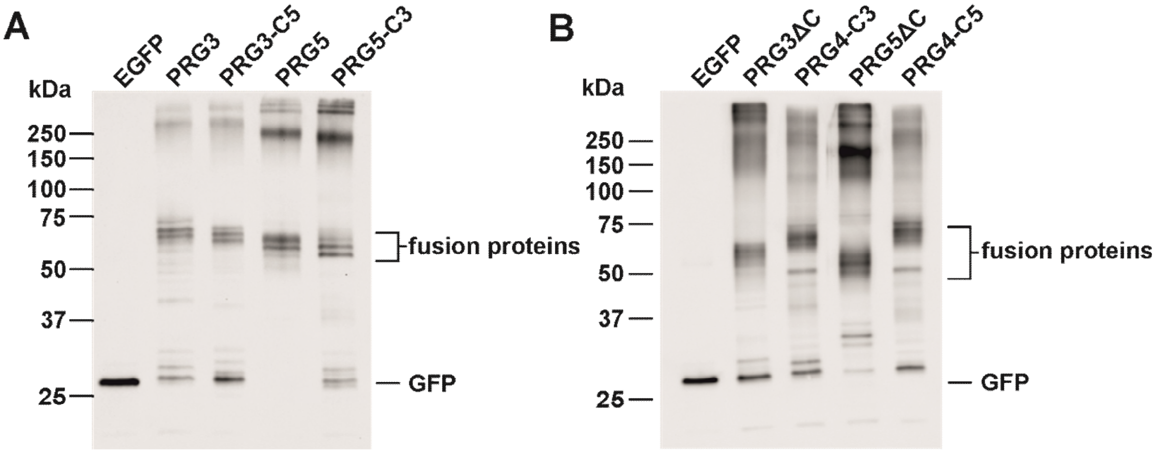

Supplementary Figure S1. Western blot analysis of fusion protein expression. The immunoblots were probed with an anti-GFP monoclonal antibody. (A) Examination of recombinant purified protein lysates of HEK293H cells expressing pEGFP-N1 alone (empty vector, control) or PRG3-, PRG3-C5-, PRG5- or PRG5-C3- EGFP fusion proteins (see T**able 5)** in immunoblot (IB) confirmed that the plasmids used in this study were sufficiently expressed. Double bands at ~63 kDa (PRG3 and PRG3-C5) and ~62.9 kDa (PRG5 and PRG5-C3) exhibited anti-GFP signal; a single band at 26.9 kDa represented GFP alone. The anti-GFP antibody also detects bands above 150 kDa in case of PRG5 and PRG5-C3 (B) Analysis of recombinant purified protein lysates expressing PRG3ΔC-, PRG4-C3-, PRG5ΔC- and PRG4-C5 (see **Table 5**) in immunoblot also confirmed sufficient expression. The deletion constructs PRG3ΔC and PRG5ΔC showed bands at ~57 kDa. PRG4-C3 and PRG4-C5 were found as double bands at ~ 63 kDa. Likewise, a band above 150 kDa is shown for PRG5ΔC. As a control, no band can be seen at ~63 kDa or ~57 kDa in EGFP transfected control cells. These results indicate expression of all plasmids in HEK293H cells.

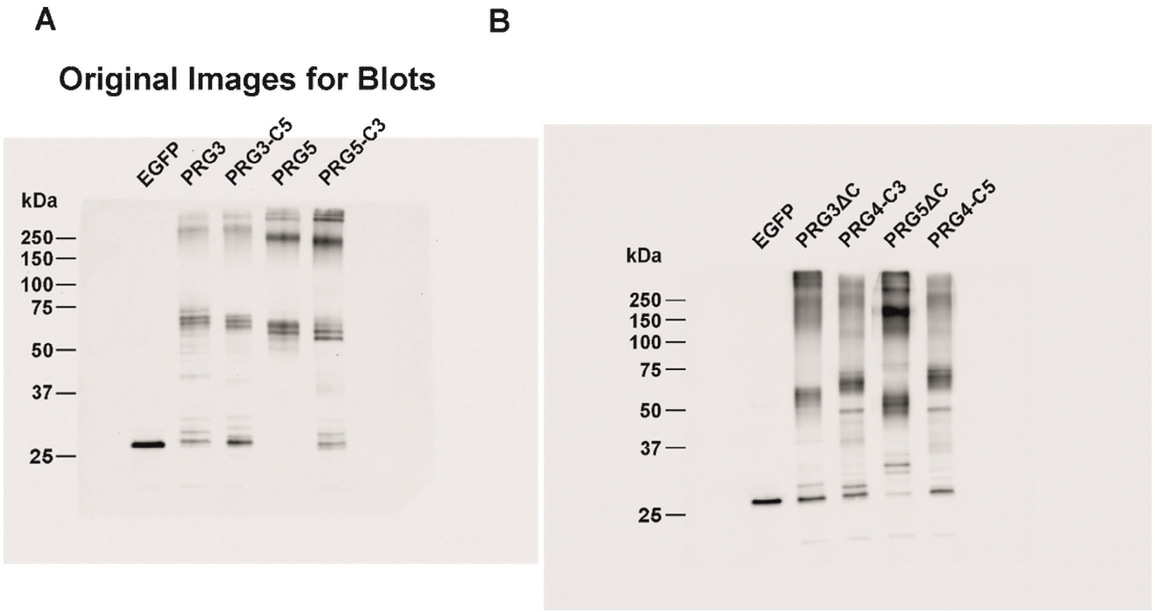

Supplementary Figure S2

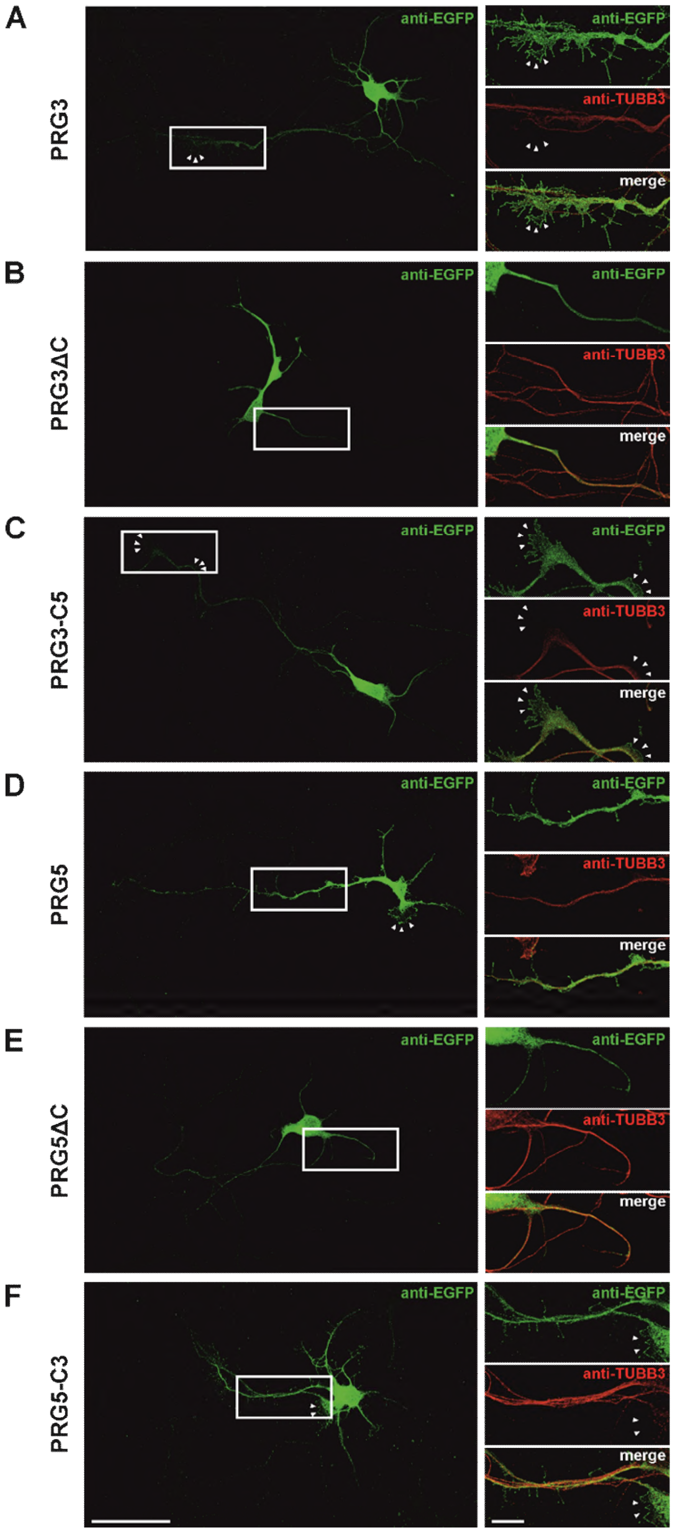

Supplementary Figure S2. Confocal stacks of representative hippocampal neurons with immunocytochemistry of the respective plasmids (PRG3, PRG3ΔC, PRG3-C5, PRG5, PRG5ΔC, PRG5-C3) stained for GFP to visualize the morphology of the cell or tubulin are shown here. Left panel: an overview of the neurons used for the higher magnification in Figure 3A, 4A is shown here. White rectangles indicate magnified areas shown in the right panels. Arrowheads denote areas showing membrane localization. Scale bars represent 50 µm (left panel) and 10 µm (right panel).

Supplementary Figure S3

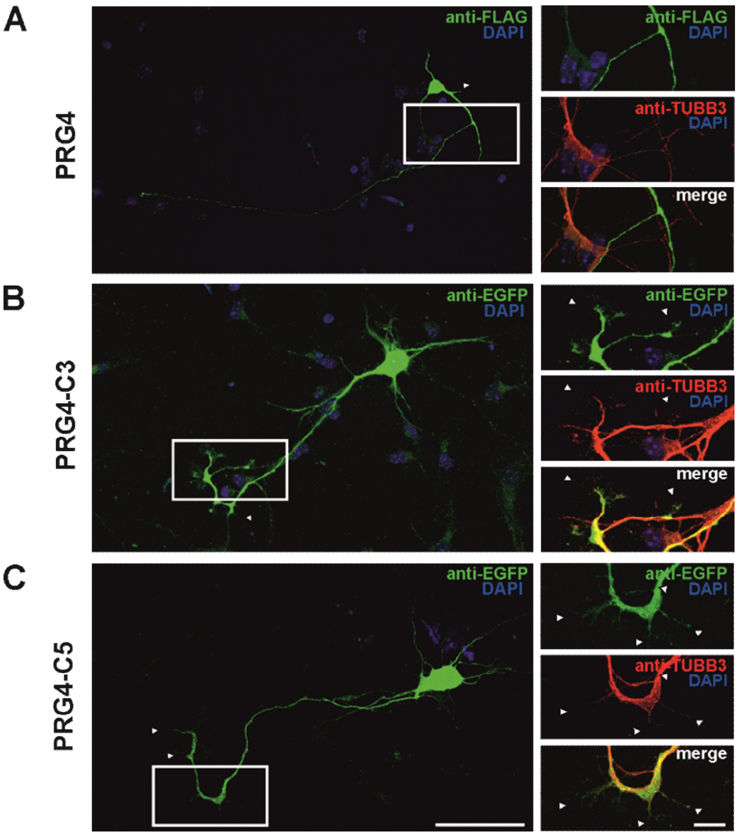

Supplementary Figure S3. Confocal stacks of representative hippocampal neurons with immunocytochemistry of the respective plasmids (PRG4, PRG4-C3, PRG4-C5) and stained for FLAG (PRG4) or GFP (PRG4-C3, PRG4-C5) to visualize the morphology of the cell or tubulin are shown here. Left panel: an overview of the neurons used for the higher magnification in Figure 6A, 7A is shown here. White rectangles indicate magnified areas shown in the right panels. Arrowheads denote areas showing membrane localization. Scale bars represent 50 µm (left panel) and 10 µm (right panel).

Supplemental material S1

Table S1: Median/IQR of data presented in **Fig. 3B-G** from N=3 independent experiments

**Condition**	**PRG3 (n=40)**	**PRG3∆C (n=41)**	**PRG3-C5 (n=38)**
		**Median/IQR**	
Number of neurites (>10 µm) per neuron	15/8	9/4	14/7
Number of major neurite length per neuron	245.6/132,1	101.7/96,4	213.8/152,4
Number of branching points per neuron	9.0/9.8	3.0/4.5	7.0/6.0
Number of short protrusions (0.2 - <2 µm) per 100 µm length per neuron	6.7/3.8	1.3/2.6	5.9/4.6
Number of medium protrusions (2 - <5 µm) per 100 µm length per neuron	4.1/1.9	0.8/1.2	3.3/1.5
Number of long protrusions (5 - <10 µm) per 100 µm length per neuron	1.2/0.8	0.6/0.6	1.0/1.0

Table S2: Median/IQR of data presented in **Fig. 4B-G** from N=3 independent experiments

**Condition**	**PRG5 (n=44)**	**PRG5∆C (n=37)**	**PRG5-C3 (n=41)**
		**Median/IQR**	
Number of neurites (>10 µm) per neuron	12.0/7.0	12.0/5.0	11.0/7.0
Number of major neurite length per neuron	171.5/121.1	139.2/89.8	194.0/152.3
Number of branching points per neuron	6.0/4.8	4.0/4.0	5.0/5.0
Number of short protrusions (0.2 - <2 µm) per 100 µm length per neuron	8.4/4.2	3.2/2.3	7.5/3.9
Number of medium protrusions (2 - <5 µm) per 100 µm length per neuron	4.1/2.5	1.5/1.4	3.1/1.9
Number of long protrusions (5 - <10 µm) per 100 µm length per neuron	1.4/1.3	0.7/0.6	1.5/0.9

Table S3: Median/IQR of data presented in **Fig. 6B-G** from N=3 independent experiments

**Condition**	**PRG4 (n=30)**	**PRG3 (n=30)**	**PRG4-C3 (n=30)**
		**Median/IQR**	
Number of neurites (>10 µm) per neuron	7.0/4.0	10.0/9.0	9.0/6.3
Number of major neurite length per neuron	114.7/114.7	117.3/116.5	169.0/155.2
Number of branching points per neuron	2.5/3.3	3.5/5.3	3.0/4.8
Number of short protrusions (0.2 -<2 µm) per 100 µm length per neuron	0.0/0.0	0.1/0.9	0.0/0.5
Number of medium protrusions (2 -<5 µm) per 100 µm length per neuron	0.0/0.5	0.9/2.3	0.0/1.1
Number of long protrusions (5 - <10 µm) per 100 µm length per neuron	0.3/0.5	0.9/1.5	0.2/1.0

Table S4: Median/IQR of data presented in Fig. **7B-G** from N=3 independent experiments

**Condition**	**PRG4 (n=30)**	**PRG5 (n=30)**	**PRG4-C5 (n=30)**
		**Median/IQR**	
Number of neurites (>10 µm) per neuron	7.0/4.0	14.0/7.8	10.0/7.8
Number of major neurite length per neuron	114.7/114.7	154.0/124.9	179.3/180.6
Number of branching points per neuron	2.5/3.3	7.5/5.8	3.0/5.0
Number of short protrusions (0.2 -<2 µm) per 100 µm length per neuron	0.0/0.0	2.7/2.4	0.3/2.5
Number of medium protrusions (2 -<5 µm) per 100 µm length per neuron	0.0/0.5	3.1/3.0	0.62/1.8
Number of long protrusions (5 -<10 µm) per 100 µm length per neuron	0.3/0.5	1.5/1.1	0.3/0.8

Table S5: Summary of oligonucleotides used for PCR and DNA sequencing

**Denotation**	**Sequence**	**Application**	**NCBI**
mPRG3ΔC-N1-pEGFP fwd	5' – GCT ACC GGA CTC AGA TCTCGA GAT G – 3'	Cloning of mPRG3ΔC into pEGFP-N1	NM_1787 56.4
mPRG3ΔCpEGFP-N1 rev	5'– AAG GAT CCG CAT GAA CCA CAC ACA TTC C – 3'	Cloning of mPRG3ΔC into pEGFP-N1	NM_1787 56.4
rPRG5ΔC- N1-pEGFP fwd	5' – GAT CTC GAG CTC AAG CTTCGA ATT CGC C – 3	Cloning of rPRG5ΔC into pEGFP-N1	NM_0011 07720.1
rPRG5ΔC-pEGFP-N1 rev	5'– AAG GAT CCG CGA AGT TAT TTA CCA CGC ATA C – 3'	Cloning of rPRG5ΔC into pEGFP-N1	NM_0011 07720.1
Fwd-pFlagPRG4	5'-GAC CTC GAG AAG CTT ATG GCT-3'	Cloning of pEGFP-N1-mPRG4ΔC-C3	NP_001277228.1
Rev-pFlagPRG4	5'-CAC TGG AGT GGC AAC TTC CA-3'	Cloning of pEGFP-N1-mPRG4ΔC-C3	NP_001277228.1
pEGFP-N1 for	5'– GTC GTA ACA ACT CCG CCC – 3'	Sequencing primer	U55762.1
pEGFP-N1 rev	5'– GTC CAG CTC GAC CAG GAT G – 3'	Sequencing primer	U55762.1

Table S6: Primary antibodies and concentrations used for immunocytochemistry and western blot experiments

**Antibody**	**Dilution for Immunocytochemistry**	**Dilution for Western Blot**	**Source**	**Identifier**
Anti-GFP (JL-8)	1:1000	1:2500	Clontech/Takara Holdings Shimogyo-ku, Kyoto, Japan	Cat#632380 RRID:AB_10013427
Anti-FLAG (HM-2)	1:1000	-	Sigma-Aldrich Chemie, Steinheim, Germany	Cat#M4403 RRID:AB_477193
Anti-ß-III Tubulin Tuj1 (TUBB3)	1:1000	-	Merck Millipore Billerica, Massachusetts, USA	Cat#AB9354 RRID:AB_570918

Table S7: Secondary antibodies/fluorescent stains and concentrations used for immunocytochemistry and western blot experiments

**Antibody/fluorescent stain**	**Dilution for Immunocytochemistry**	**Dilution for Western Blot**	**Source**	**Identifier**
Goat-anti-mouse Alexa Fluor 488	1:1000	-	Thermo Fisher Scientific, Waltham, MA, USA	Cat#A11001
Goat-anti-chicken IgY Alexa Fluor 568	1:1500	-	Thermo Fisher Scientific	Cat#A11077
Rabbit-anti-chicken TRITC	1:400	-	Sigma-Aldrich Chemie	Cat#T6903
Phalloidin-iFluor 555	1:1000	-	Abcam, Cambridge, UK	Cat#ab176756
DAPI (1 mg/ml)	1:1000/1:2000	-	Sigma-Aldrich Chemie	Cat#D9542
Sheep-anti-Mouse IgG HRP	-	1:10000	GE Healthcare, Solingen, Germany	Cat#NA931
